# Proteomics of *Neisseria gonorrhoeae*: the treasure hunt for countermeasures against an old disease

**DOI:** 10.3389/fmicb.2015.01190

**Published:** 2015-10-26

**Authors:** Benjamin I. Baarda, Aleksandra E. Sikora

**Affiliations:** Department of Pharmaceutical Sciences, College of Pharmacy, Oregon State University, Corvallis, OR, USA

**Keywords:** *Neisseria gonorrhoeae*, gonorrhea, proteomics, molecular targets, vaccine, drugs, antibiotic resistance, surveillance

## Abstract

*Neisseria gonorrhoeae* is an exquisitely adapted, strictly human pathogen and the causative agent of the sexually transmitted infection gonorrhea. This ancient human disease remains a serious problem, occurring at high incidence globally and having a major impact on reproductive and neonatal health. *N. gonorrhoeae* is rapidly evolving into a superbug and no effective vaccine exists to prevent gonococcal infections. Untreated or inadequately treated gonorrhea can lead to severe sequelae, including pelvic inflammatory disease and infertility in women, epididymitis in men, and sight-threatening conjunctivitis in infants born to infected mothers. Therefore, there is an immediate need for accelerated research toward the identification of molecular targets for development of drugs with new mechanisms of action and preventive vaccine(s). Global proteomic approaches are ideally suited to guide these studies. Recent quantitative proteomics (SILAC, iTRAQ, and ICAT) have illuminated the pathways utilized by *N. gonorrhoeae* to adapt to different lifestyles and micro-ecological niches within the host, while comparative 2D SDS-PAGE analysis has been used to elucidate spectinomycin resistance mechanisms. Further, high-throughput examinations of cell envelopes and naturally released membrane vesicles have unveiled the ubiquitous and differentially expressed proteins between temporally and geographically diverse *N. gonorrhoeae* isolates. This review will focus on these different approaches, emphasizing the role of proteomics in the search for vaccine candidates. Although our knowledge of *N. gonorrhoeae* has been expanded, still far less is known about this bacterium than the closely related *N. meningitidis*, where genomics- and proteomics-driven studies have led to the successful development of vaccines.

## Introduction

Gonorrhea is an ancient human disease, with references to its symptoms found in the Old Testament of the Bible (Leviticus 15:1–3). For almost 700 years, it has been known as “the clap,” a likely reference to the old Le Clapiers district of Paris where prostitutes were housed. This sexually transmitted disease remains a global scourge today, causing an estimated 106 million new cases worldwide each year WHO (2012). In the United States, gonorrhea is the second most common sexually transmitted disease, with over 300,000 new cases, primarily affecting those between 20 and 24 years of age, reported to the Centers for Disease Control and Prevention (CDC) annually CDC (2013). The Gram-negative diplococcus *Neisseria gonorrhoeae*, the gonococcus (GC), is the sole cause of gonorrhea. In men, infections typically present as profuse, localized inflammatory response of the urethra (i.e., urethritis). In contrast, gonorrhea remains asymptomatic in 50–80% of infected women (Farley et al., 2003; WHO, 2011). Untreated or inadequately treated gonococcal infections can have severe consequences including epididymitis in men and pelvic inflammatory disease and inflammation of the uterus, ovaries and fallopian tubes in women, which can lead to infertility. Neonatal health is also detrimentally affected by GC infection, as this pathogen can cause a sight-threatening conjunctivitis in infants born to infected mothers (Creighton, 2011). Additionally, infection with GC increases the risk of HIV transmission (Tapsall, 2005). Further compounding the difficulty in treating gonorrheal infections, through a number of point mutations, as well as horizontally acquired genes, GC has gained resistance to nearly all antibiotics currently in use (Tapsall et al., 2010; Unemo and Shafer, 2014). The CDC now recommends a combination of ceftriaxone with either doxycycline or azithromycin for empirical gonorrhea treatment (CDC, 2012b); however, treatment failures with ceftriaxone have been verified in Japan, Australia, Sweden, and Slovenia (reviewed in Unemo, 2015). Additionally, GC demonstrates remarkable heterogeneity and strain-to-strain variability, which represent a significant challenge in vaccine development (Zhu et al., 2011; Jerse et al., 2014).

Immediate action is critically needed against gonorrhea before it becomes completely untreatable. In response to this dire possibility, the World Health Organization (WHO) published the “Global Action Plan to Control the Spread and Impact of Antimicrobial Resistance in *Neisseria gonorrhoeae*” (WHO, 2012), and the CDC (CDC, 2012a), as well as the European Centre for Disease Prevention and Control (ECDC; ECDC, 2012) proposed region-specific response plans. Overall, these proposals stressed the importance of implementing holistic action against gonorrhea, which would encompass early prevention, diagnosis, contact tracing, treatment, and surveillance of antimicrobial resistance and treatment failures.

**FIGURE 1:**
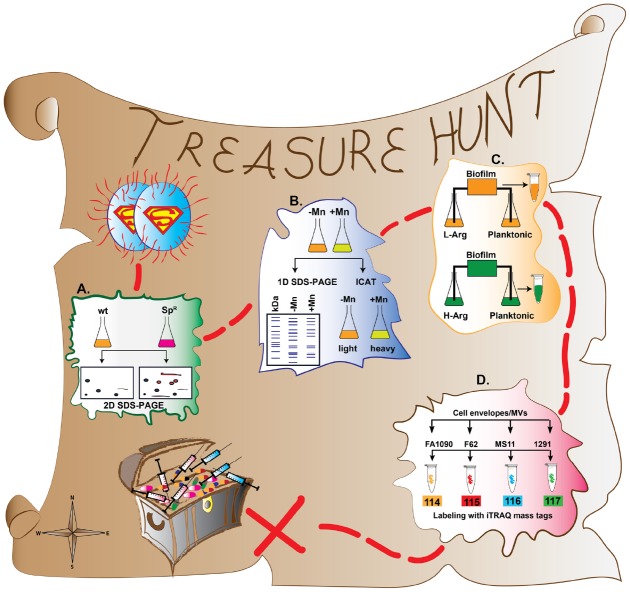
**A treasure hunt map of proteomic approaches applied to better understand GC pathophysiology and discovery of vaccine and drug targets. (A)** Comparative proteome maps have been constructed between spectinomycin resistant (Sp^R^) and spectinomycin sensitive (wild type; wt) GC isolates using 2-D-SDS-PAGE. **(B)** Proteomic investigations (1D SDS-PAGE and ICAT) of manganese (Mn) regulation of virulence factors and oxidative stress. **(C)** Proteomic profiling of GC transition from planktonic to biofilm growth using SILAC. **(D)** Comparative, high-throughput proteomic analyses of cell envelope and MV fractions derived from GC strains FA1090, F62, MS11, and 1291.

An ideal method of gonorrhea prevention would be the development of a protective vaccine(s). Indeed, according to model simulations, gonococcal prevalence could be reduced by at least 90% after 20 years if all 13-year olds were given a non-waning vaccine with 50% efficacy or a vaccine with 100% efficacy that wanes after 7.5 years. Further, even with a non-waning vaccine of 20% efficacy, as much as a 40% decrease in prevalence could be anticipated (Craig et al., 2015). In line with the WHO, CDC, and ECDC call for action, it is a prerequisite that the anti-gonorrhea vaccine and new drug development be made a priority. Different proteomic approaches are exceedingly valuable to accompany the progress of effective new therapeutic interventions by identifying vaccine and drug targets. Herein, we guide through these different approaches in the treasure hunt for countermeasures against gonorrhea, emphasizing the role of proteomics in the search for GC vaccine candidates (Figure 1).

## How Does a Health-Obsessed Researcher Evaluate an Energy Bar?

## With Proteomics

As genomic approaches, whole-genome sequencing in particular, have become relatively inexpensive and increasingly high-throughput, with short turn around times and great resolution in the past few years, they have grown increasingly useful in basic research and in clinical diagnosis. The Broad Institute has recently released the whole genome sequences of 14 GC clinical isolates in collaboration with the *Neisseria gonorrhoeae* Group to facilitate research into pathogenesis and genetic determinants of disease states (*Neisseria gonorrhoeae* Group Sequencing Project, Broad Institute of Harvard and MIT^1^). Additionally, multilocus sequence typing (MLST), which characterizes isolates based on internal fragments of housekeeping gene alleles, has been used to cluster GC patient isolates based on phenotype (Ilina et al., 2010). A database of MLST data for *Neisseria* species has been established^2^, further facilitating MLST identification and genotypic grouping of GC isolates. Genomic-derived methodologies have identified GC iron-responsive genes (Ducey et al., 2005), the anaerobic stimulon (Isabella and Clark, 2011), as well as gene expression patterns during infection of the lower female genital tract (McClure et al., 2015). In clinical applications, the proliferation of genomic datasets has promoted the development of nucleic acid amplification tests (NAATs), which allow for rapid identification of GC in patient samples without the need for culture (Low et al., 2014).

Ultimately, however, genomics is unable to capture the complete biological complexity present. A combination of different proteomic approaches can greatly complement genomic-acquired data by examining the GC protein population in biofilms or upon exposure to relevant host stimuli (Wu et al., 2010; Phillips et al., 2012), proteins associated with drug resistance (Nabu et al., 2014), proteome expression patterns during infection, post-translational modifications, or by providing information about proteins’ subcellular location, structures and protein–protein interactions. The knowledge gained from proteomic studies can be useful for identifying GC in clinical samples (Gudlavalleti et al., 2008; Carannante et al., 2015), evaluating antibiotic resistance, and discovering potential vaccine and drug candidate proteins (Zielke et al., 2014, 2015).

## When do you not want to Receive Applause?

## When you Identify “The Clap”

Rapidly identifying GC in clinical isolates is vital to initiate treatment as quickly as possible to prevent the severe consequences of untreated gonorrhea, as well as to limit the spread of antimicrobial resistant strains. NAATs, developed with the use of genomic data, are more rapid and sensitive than culture, which has resulted in an increase in the number of infections detected (Unemo and Shafer, 2014; Wind et al., 2015). Current commercially available gonococcal NAATs are unable to satisfactorily measure antimicrobial resistance, undermining the surveillance of antimicrobial resistance trends. Because of this limitation, the ECDC has recommended that all specimens positive for GC by NAAT are subsequently cultured to monitor antimicrobial resistance trends (ECDC, 2012). However, laboratory-developed NAATs have been utilized to identify known genetic antimicrobial resistance determinants against several classes of antibiotics as well as detecting the crucial mutations conferring resistance to extended-spectrum cephalosporins in superbug strains H041 and F89 (reviewed, by Unemo, 2015).

As antibiotic resistance determinants continuously evolve, the development of new detection tests is required (Unemo, 2015). Proteomics can provide sensitive, accurate, rapid, and cost-effective methods of GC identification and determination of antimicrobial resistance patterns in clinical samples. Proteomic identification of bacteria is primarily performed with data generated by matrix-assisted laser desorption ionization time-of-flight mass spectrometry (MALDI-TOF-MS). Unique and representative biomarker ions can be established from intact cell MALDI-TOF-MS analysis (van Baar, 2000; Amiri-Eliasi and Fenselau, 2001; Fenselau and Demirev, 2001; Fagerquist et al., 2010; Murray, 2010; Niyompanich et al., 2014). The most important advantages in direct bacterial profiling by means of MALDI-TOF-MS are: (1) the requirement for only a small amount of biological material, (2) the possibility of examining intact cells without preceding extraction and separation, (3) a fast and straightforward procedure, and (4) high specificity in species differentiation (Ilina et al., 2009).

One of the first examples of direct GC profiling with MALDI-TOF-MS used surface enhanced MALDI-TOF-MS to analyze over 350 GC strains and closely related species (Schmid et al., 2005). These comparisons enabled the design of multilayer artificial neural networks that revealed 20 ion peak descriptors of positive, negative and secondary nature that were supreme for GC identification (over 96% efficiency, a sensitivity of 95.7% and a specificity of 97.1%). Another study used atmospheric pressure MALDI-TOF to determine that a putative DNA binding protein from *N. meningitidis*; its homolog in GC, DbhA; and acyl carrier proteins in each species could be used as protein biomarkers for identifying pathogenic *Neisseria* (Gudlavalleti et al., 2008). Although this approach does not provide a method to distinguish between *N. meningitidis* and *N. gonorrhoeae*, it can act as a base upon which to build techniques for identifying pathogenic *Neisseria* in clinical samples. The successful application of MALDI-TOF-MS for GC identification has also been recently demonstrated on 92 out of 93 isolates of gonococci collected from 2007 to 2012 as part of the European Gonococcal Antimicrobial Surveillance Programme (Carannante et al., 2015).

Together, these studies highlight the potential of proteomic approaches to rapidly and correctly identify GC in various clinical isolates, which, if implemented on a larger scale, will promote rapid initiation of treatment while still allowing antimicrobial susceptibility testing to be performed.

## What did the Bacteria Call their Guerilla Warfare Unit?

## The Spectinomycin Resistance

In a survey of antimicrobial resistance in Southeast Asia, the WHO Global Gonococcal Antimicrobial Surveillance Program found that between 0.6 and 10.5 percent of isolates demonstrated spectinomycin resistance (Bala et al., 2013). Spectinomycin directly interacts with 16S rRNA and inhibits the elongation factor G (EF-G)-catalyzed translocation of the peptidyl-tRNA from the A site to the P site during protein synthesis (Bilgin et al., 1990; Ramakrishnan and White, 1992). Not surprisingly, spectinomycin resistance determinants traditionally involve mutations in 16S rRNA (Maness et al., 1974; Galimand et al., 2000). However, a deletion of amino acid 25 and a K26E amino acid alteration (*E. coli* numbering) in the ribosomal protein S5 is a newly identified mechanism associated with high-level spectinomycin resistance in GC (Unemo et al., 2013). Overall, however, the *in vitro* susceptibility to this aminocyclitol compound is remarkably high worldwide and this antibiotic is an effective alternative for treatment of anogenital gonorrhea, in particular for multidrug resistant cases (Unemo, 2015).

The proteomic signatures of GC spectinomycin resistance as well as cellular responses to spectinomycin treatment were assessed through qualitative and relative quantitative proteomics using densitometry analysis of proteins separated by two-dimensional sodium dodecyl sulfate-polyacrylamide gel electrophoresis (2D SDS-PAGE) and protein identification with MALDI-TOF-MS (Otto et al., 2012; Nabu et al., 2014). Comparative proteome maps have been constructed between a spectinomycin resistant (Spec^R^) clinical isolate and a spectinomycin sensitive (Spec^S^) reference strain (ATCC 49226; Figure 1A). When the two strains were not exposed to spectinomycin, their protein profiles were largely the same, with EF-Tu and EF-Ts, cysteine synthase, and the septum site-determining protein MinD upregulated in the Spec^R^ isolate. Additionally, MinD, oxidoreductase, and hypothetical protein NGO1873 were shifted to a more acidic isoelectric point, and the ribosomal protein S6 was shifted to a more basic pI in the resistant isolate. Finally, ABC transporter substrate-binding protein showed decreased expression and alcohol dehydrogenase was not detected in the Spec^R^ strain. Interestingly, in the presence of spectinomycin, although dihydrolipoamide dehydrogenase, peroxiredoxin, an outer membrane protein Rmp, and the 50S ribosomal protein L7/L12 were upregulated in both strains, none of the proteins were expressed as highly in the resistant strain as in the wild type. Overall, the spectinomycin treatment of the Spec^S^ GC resulted in alterations in the abundance of proteins involved in energy metabolism and detoxification, as well as cell envelope proteins (Rmp and ABC transporter substrate-binding protein).

Based on the obtained proteomic comparisons, a mode of spectinomycin action on GC is proposed but not experimentally validated (Nabu et al., 2014). Briefly, in the presence of sub-minimal inhibitory concentrations, the drug destabilizes the outer membrane, recruiting Rmp to maintain the integrity of the cell envelope. Spectinomycin accumulates in the periplasm, generating a concentration imbalance between the inside and outside of the cell. In response, significant changes in energy metabolism including an increase in NADH production and oxidation through the electron transport chain occur. At the same time, additional proton motive force allows more drug molecules to enter the cell. Increased NADH oxidation leads to an increase in reactive oxygen species, which are detoxified by the increase in peroxiredoxin and glutamate dehydrogenase production. Higher levels of L7/L12 allow the cell to overcome the inhibition of ribosomal translocation imposed by spectinomycin. In the Spec^R^ strain, amino acid ATP binding cassette transporter substrate-binding protein levels are decreased even in the absence of spectinomycin, possibly affecting uptake of the drug. Expression of enolase is decreased, which may result in increased levels of 2P-D-glycerate and 3P-D-glycerate. Together with an increase in cysteine synthetase expression, this may improve the cell’s defense against reactive oxygen species. Finally, the already high levels of EF-Tu and –Ts, together with the upregulation of L7/L12, may assist with protein translation in the presence of spectinomycin (Nabu et al., 2014).

Future research may be able to exploit some of the suggested secondary pathways and increase the efficacy or reduce the resistance potential of future antimicrobial compounds. Also apparent in this study are some of the shortcomings of 2D SDS-PAGE, which has limited utility when dealing with low abundance and/or membrane proteins.

## How did the Health-Obsessed Researcher Compare two Protein Shakes?

## With Quantitative Proteomics

In contrast to qualitative proteomics, quantitative proteomic approaches allow for (absolute or relative) quantification of proteins on a global scale. The object of quantitative proteomics is to identify specific alterations between control samples and particular experimental conditions (e.g., healthy versus diseased state). In addition, quantitative proteomic profiling may focus on a specific subset of proteins (subproteome), where for instance bacterial whole cell lysates are subjected to fractionation to enrich for cell envelope proteins. Very often the proteins of interest are relatively low in abundance; therefore it is critical to utilize appropriate combinations of pre-fractionation techniques such as different kinds of liquid chromatography (affinity, reversed phase, size-exclusion, or ion exchange) to reduce the complexity of analyzed samples, in addition to employing sensitive mass spectrometry instruments. Different approaches have been developed to facilitate quantitative proteome profiling studies involving stable isotope labeling, such as ICAT, ICPL, SILAC, iTRAQ, TMT, and IPTL. In addition, label-free statistical methodologies (MRM, SWATH) and absolute quantification methods by mass spectrometry (AQUA strategy) have become available (reviewed in Chahrour et al., 2015).

Quantitative proteomic approaches, involving different stable isotope labels and summarized in Table 1, have been used to investigate how GC responds to oxidative stress (Wu et al., 2010), transitions from planktonic to biofilm growth (Phillips et al., 2012), and adapts the composition of the cell envelope in response to different environmental cues encountered in micro-ecological niches within the host (Zielke et al., 2015). In addition, iTRAQ technology has also revealed the dynamic subproteomes of cell envelopes and naturally released membrane vesicles in four different GC isolates (Zielke et al., 2014).

**TABLE 1:** **Quantitative Proteomic Approaches utilized to studying GC**.

**Proteomic Approach**	**Principles**	**Use in GC**	**References**
Isotope coded affinity tag (ICAT)	- Post-harvest labeling - Heavy or light isotopic reactive groups label cysteine residues in a protein sample. - Two samples are pooled and analyzed by MS. - Relative quantification of proteins by comparing heavy: light ratio.	Response to oxidative stress	Wu et al. (2010)
Stable isotope labeling by amino acids in cell culture (SILAC)	- Metabolic labeling - Growth medium is supplemented with either light (unlabeled) or heavy (labeled) amino acid. - Two samples are pooled and analyzed by MS. - Relative quantification of proteins by comparing heavy: light ratio	Shift from planktonic to biofilm growth	Phillips et al. (2012)
Isobaric Tagging for Relative and Absolute Quantification (iTRAQ)	- Post-harvest labeling - Proteolytically digested proteins are tagged on N-termini and lysine side chains with isobaric mass labels. - Two to eight samples are pooled and analyzed by MS. - Relative or absolute quantification of proteins by measuring intensity of reporter ion peak.	Cell envelopes and membrane vesicles Comparison of cell envelope profiles upon environmental cues encountered in the host	Zielke et al. (2014); Zielke et al. (2015)

## What Happens when two Oxen go on their First Date?

## Oxidative Stress

In a typical inflammatory response to GC, neutrophils are recruited to the site of infection by chemokines, such as IL-8, released by infected mucosal surfaces (Criss et al., 2009). When activated neutrophils phagocytize GC, the production of reactive oxygen species (ROS) is either stimulated or inhibited, depending on GC expression of opacity-associated proteins (Opa). Cells expressing Opa protein (Opa^+^) ligate to human carcinoembryonic antigen-related cell adhesion molecules (CEACAMs), specifically CEACAM3, on the surface of the neutrophil, triggering phagocytosis, NADPH subunit assembly, and degranulation (Schmitter et al., 2007; Sarantis and Gray-Owen, 2012). Additionally, Opa_57_ protein ligated to CEACAM3 amplifies the inflammatory response by activating nuclear factor (NF)-κB and increasing phosphorylation of the p38 kinase (Sintsova et al., 2014).

This cascade of ROS production puts the GC cells under tremendous oxidative stress. However, viable GC has been shown to survive and replicate within neutrophils, even after NADPH oxidase activation (Simons et al., 2005), indicating that GC is able to protect itself against oxidative stress. This pathogen can defend against superoxide radicals (Tseng et al., 2001) and hydrogen peroxide (Seib et al., 2004) in a manganese (Mn) dependent manner. A study combining transcriptomic and qualitative- and quantitative-proteomic approaches examined the protective mechanism utilized by GC in the presence of Mn (Wu et al., 2010). One-dimensional sodium dodecyl sulfate-polyacrylamide gel electrophoresis (1D SDS-PAGE) paired with one-dimensional liquid chromatography-tandem mass spectrometry (1D LC-MS/MS) identified 46 proteins expressed only in the presence of Mn. Notably, bacterioferritin, azurin (Laz), and iron-superoxide dismutase (SodB)—proteins involved in defense against oxidative stress—were expressed in bacteria cultured in the presence of Mn, but were not detected in those cultured without Mn. When cultured in the absence of Mn, GC was found to express an outer membrane methionine sulfate reductase that is also involved in defense against superoxide and hydrogen peroxide.

Further, a quantitative proteomic investigation using Isotope-Coded Affinity Tag (ICAT) labeling was used to determine which proteins were differentially regulated by the presence or absence of Mn (Wu et al., 2010). For ICAT analysis, two protein samples are labeled with reactive groups (biotinylated iodoacetamide or acrylamide derivatives) that specifically react with the sulphydryl groups of denatured peptides’ cysteine side chains (Gygi et al., 1999). One sample is labeled with a light isotope, while the other sample is labeled with a heavy isotope. The samples are then combined and analyzed by mass spectrometry (Figure 1B; Table 1). The protein populations can then be quantified by comparing the ratio of heavy to light proteins (Gygi et al., 1999; Colangelo and Williams, 2006; Chahrour et al., 2015).

In this study, ICAT labeling, coupled with MS/MS, revealed numerous proteins that were downregulated more than 1.5-fold in the presence of Mn, including PilT (an ATPase involved in pilus disassembly), OmpR, a 64 KDa outer membrane protein (OMP P64k), and peroxiredoxin, which reduces and detoxifies peroxides (Seib et al., 2006). Additionally, Mn affected the levels of pilin, superoxide dismutase, and pyrophosphatase without causing a corresponding change in the transcript level, indicating that these proteins are likely to be regulated post-transcriptionally by the presence of Mn (Wu et al., 2010).

Taken together, the results of this study suggest that, in the presence of Mn, GC upregulates the expression of iron storage proteins that protect against oxidative damage. Concomitantly, the bacterium downregulates pyrophosphatase (Ppa) and polyphosphate kinase (Ppk). Ppa hydrolyzes inorganic pyrophosphate (PP_i_) into two molecules of orthophosphate (P_i_), while Ppk synthesizes polyP from P_i_. When these two proteins are downregulated, PP_i_ accumulates in the cell and chelates Mn. In a Mn(II)-PP_i_ complex, Mn is able to defend against ROS non-enzymatically (Wu et al., 2010).

The information gleaned from these proteomic experiments could suggest a method to combat gonorrhea whereby the downregulation of protective proteins required only in the absence of Mn is maintained, but the alteration of the levels of proteins required for protection from oxidative stress in the presence of Mn is blocked. Importantly, this study highlights the utility of proteomic approaches to investigate biological responses involving post-transcriptional regulation that genomic methods alone cannot discover. It is important to keep in mind, however, that ICAT has two major drawbacks: (1) proteins that do not have any cysteine residues will be eliminated from this analysis, and (2) the release of biotinylated peptides from the streptavidin column is not quantitative for low-abundance peptides (Chahrour et al., 2015).

## What is a Microbiologist’s Favorite kind of Movie?

## A Biofilm

Bacteria often shift from planktonic (free living bacteria) growth to a biofilm community, where bacteria grow in close proximity to each other, protected by an extracellular polymer composed of polysaccharides, proteins, nucleic acids, and lipids (Flemming and Wingender, 2010). GC has been shown to form biofilms *in vitro* (Greiner et al., 2005), and an examination of primary cervical epithelial cells from cervical biopsy samples revealed biofilm growth in culture positive gonorrhea cases (Steichen et al., 2008). Biofilms exacerbate antibiotic resistance by providing a protective barrier against antimicrobial action, and biofilms formed by GC are thought to contribute to asymptomatic infections (Steichen et al., 2008).

To understand the mechanisms underlying biofilm formation, a quantitative proteomic study examined the proteome changes GC undergoes in the transition from planktonic to biofilm growth using stable isotope labeling by amino acids in cell culture (SILAC; Figure 1C; Table 1; Phillips et al., 2012). One of the important advantages of SILAC over other stable isotope labeling methods is that the label is integrated into the peptide at early stages of experimentation when the sample is metabolically active. Thus, possible variability due to sample preparation and purification losses are eliminated (Chahrour et al., 2015).

In this analysis, planktonic cells of GC strain 1291, which is an arginine auxotroph, were grown with labeled ^13^C_6_-Arg, and biofilm cells were grown with unlabeled Arg in a continuous-flow apparatus. Extracted protein samples from each bacterial population were combined and subjected to MALDI-TOF mass spectrometry analysis. Overall, this global analysis identified 757 proteins, 152 of which were significantly differentially expressed. In particular, GC cultured in a biofilm exhibited 73 upregulated- and 54 downregulated-proteins when compared to planktonic growth. The results of this study indicated that the bacteria upregulate proteins to respond to an oxygen-limited environment, including cytochrome c oxidase subunit III CcoP and nitrite reductase AniA. To cope with restricted nutrient availability in the biofilm, bacterial metabolism is shifted to increase sugar fermentation and tricarboxylic acid (TCA) cycle enzymes. The composition of the outer membrane is also altered during growth in a biofilm, with increased levels of 9 proteins including OpaB and OpaD (Phillips et al., 2012), both of which have been shown to adhere to and damage fallopian tube mucosa (Dekker et al., 1990). Among the downregulated proteins in the biofilm were proteins involved in energy metabolism, protein fate and synthesis, and transport and binding proteins, specifically iron complex outer membrane receptor protein (FetA) as well as transferrin-binding protein B and A (TbpB and TbpA, respectively).

A direct comparison of the transcriptome (Falsetta et al., 2009) and proteome expression profiles of GC biofilms showed a very poor correlation with only seven overlapping hits including AniA, OpaB, cytochrome C peroxidase CcpR, putative dihydrolipoamide dehydrogenase, putative cysteine synthase/cystathionine beta-synthase, hypothetical protein NGO0905, and putative ABC transporter NGO1494 (Phillips et al., 2012).

This study gives insight into the adaptations necessary for GC to establish long-term infections and emphasizes the utility of proteomic approaches to examine these adaptations. In addition, the identified upregulated outer membrane proteins may be utilized as biomarkers for gonorrhea diagnostics.

## What Book-Like Candidate was not at the Last Presidential Debate?

## A Novel Vaccine Candidate

Perhaps one of the most exciting uses of proteomic approaches is in the search for new ways to combat multidrug resistant GC. We are applying a proteomics-driven reverse vaccinology approach to identify vaccine candidate proteins against gonorrhea (Zielke et al., 2014, 2015). Reverse vaccinology searches for possible vaccine candidate proteins using different genomics and proteomics methodologies and has already been successfully applied to different pathogenic bacteria including *N. meningitidis* serogroup B (Heckels and Williams, 2010; Adamczyk-Poplawska et al., 2011; Seib et al., 2012; Delany et al., 2013; Heinson et al., 2015).

No vaccine against GC currently exists, although research has been ongoing for decades. Two attempted vaccines, comprised of killed whole cells and purified pilin protein, failed in clinical trials over 13 years ago (Zhu et al., 2011). Since that time, very little research into gonorrheal vaccines has occurred, mainly due to the highly variable targeted surface proteins. Because GC is a strict human pathogen, research was also hampered by the lack of a suitable small animal model for gonorrheal infection (Jerse et al., 2014). Fortunately, a mouse model of female infection was developed, in which female mice are treated with 17-β-estradiol when they are in the diestrus stage of the estrus cycle. The mice are also treated with an antibiotic cocktail of streptomycin sulfate, vancomycin HCl, and trimethoprim sulfate to prevent overgrowth of commensal vaginal bacteria while under the influence of estradiol. 2 days after estradiol treatment, GC is introduced intravaginally. Using this model, GC can be recovered an average of 12.2 days post-inoculation with 10^6^ colony forming units (Jerse, 1999). A further advancement in the mouse model has been the development of transgenic mice that express human CEACAM proteins, providing a closer reproduction of conditions encountered in the human host (Jerse et al., 2014). The availability of a mouse model has greatly facilitated vaccine research. The immune response to infection, as well as resistance to subsequent infections after inoculation with an experimental vaccine can be closely monitored and investigated with the genetic tools available for studying mice (Zhu et al., 2011). However, to fully utilize this model for vaccine research, suitable candidate proteins must be identified—a goal for which proteomic approaches are ideally suited.

During the development of the MenB vaccine, out of nearly 600 candidates selected by reverse vaccinology, 350 recombinant proteins were successfully expressed in *Escherichia coli* and evaluated for their surface exposure. A total of 28 among them elicited bactericidal antibodies against Group B meningococci *in vitro*. Finally, the neisserial heparin-binding antigen NHBA, factor H-binding protein fHbp, as well as the neisserial adhesin NadA were chosen as part of the MenB vaccine (Seib et al., 2012; Delany et al., 2013; Jerse et al., 2014). In contrast, only 12 different candidates are being evaluated as potential gonorrhea vaccine antigens (Jerse et al., 2014). Therefore, a more far-reaching effort is required to make a gonorrhea vaccine a reality.

Of particular interest for vaccine development and identification of new drug targets are proteins localized to the bacterial cell envelope and membrane vesicles (MVs)—spherical outpouchings of the cell envelope—as they interact directly or indirectly with host tissues; play roles in pathogenesis, antibiotic resistance, and biofilm formation; and participate in general physiological processes. Surprisingly few studies addressed GC cell envelope composition (Yoo et al., 2007; Phillips et al., 2012; Zielke et al., 2014). Also, despite studies reporting the release of MVs and their different morphological forms (spherical, lobed, and tubular) in GC from the early 1970s, only a few reports focused on elucidating their components (Swanson et al., 1971; Dorward et al., 1989; Pettit and Judd, 1992a,b; Falsetta et al., 2011; Zielke et al., 2014).

To begin the systematic mining of GC cell envelope and MVs for the discovery of vaccine and drug candidates, we first used the PSORTb 3.0.2 (Gardy et al., 2005) bioinformatics predictions and analyzed the subcellular localization of all ORFs in the completed genome sequence of strains FA1090 (Gen Bank accession number AE004969) and NCCP11945 (Gen Bank accession number CP001050), as well as the draft genome sequences of 14 different GC strains (downloaded from the Broad Institute website^3^). These studies revealed that, on average, about 50 of the 2,000 ORFs present in the GC genome encode outer membrane proteins (Zielke and Sikora, 2014). However, the subcellular location could not be predicted for about 30% of all ORFs. This analysis demonstrated that there is still much to learn about GC cell envelope composition and opened up exciting prospects for applying proteomics for the discovery of vaccine targets.

As proteomic investigations of membrane proteins are technically challenging, we chose to apply gel-free quantitative proteomic approaches including isobaric tagging for relative and absolute quantification (iTRAQ, Table 1) combined with multidimensional liquid chromatography and tandem mass spectrometry (2D-LC/MS/MS) to examine cell envelopes and naturally released MVs (Zielke et al., 2014). Four GC strains: FA1090, F62, MS11, and 1291 were cultured in liquid media under standard growth conditions and their cell envelopes and MVs were harvested in mid-logarithmic phase of growth. iTRAQ quantification was performed by labeling proteins isolated from subproteome fractions of each strain of interest with one of four isobaric tags (N-hydroxysuccinimide ester-activated compounds) that react to free amine groups on the N-termini and lysine side chains of proteins with high efficiency (Ross et al., 2004). Each of the four tags contains a reporter ion of a unique mass (Figure 1D; Table 1). When the samples are combined and subjected to mass spectrometry, the reporter ions are released from the labeled peptide. After the release of the reporter ions, all of the identical peptides in a sample will result in identical mass spectra, and the abundance of the peptide in the four multiplexed samples can be quantified by the relative intensity of the corresponding reporter ion peak (Ross et al., 2004; Wiese et al., 2007). The advantage of using iTRAQ is that it can be easily multiplexed and up to 8 different samples can be simultaneously analyzed within a single experiment. Additionally, as the iTRAQ tags react with all primary amine functional groups of peptides, nearly all peptides are labeled and information about not only their abundance but also their modification(s) can be acquired (Chahrour et al., 2015).

Our proteomic profiling of cell envelopes and native MVs revealed 533 and 168 common proteins, respectively, in analyzed GC strains. A total of 22 differentially abundant proteins were discovered including hitherto unknown proteins. Among those proteins that displayed similar abundance in four GC strains, we identified 305 and 46 cell envelope-and MVs-associated proteins, respectively. In addition, 34 proteins were found in both cell envelopes and MVs with eleven of them differentially regulated (Zielke et al., 2014). A few of these differentially expressed proteins included cytoplasmic proteins, an observation that was confirmed by a subsequent, independent proteomic study of MVs (Perez-Cruz et al., 2015).

The ubiquitous outer membrane proteins identified included GC homologs of the outer membrane β-barrel assembly (Bam) protein complex (Ricci and Silhavy, 2012), including BamA, BamD, and BamE; lipopolysaccharide transport protein LptD; and TamA (NGO1956) and TamB (NGO1955), two proteins thought to work cooperatively to assist in the assembly of autotransporter proteins (Heinz et al., 2015). Numerous uncharacterized proteins were also ubiquitously expressed, including NGO1344, NGO1985, NGO2111, NGO2121, and NGO2139 (Zielke et al., 2014).

We further examined LptD, NGO1344, NGO1985, NGO2111, NGO2121, NGO2139, and TamB by constructing conditional-(LptD) or complete- knockout strains for each protein. These proteins were chosen because they contain domains predicted to function in maintaining cell envelope homeostasis. LptD expression in GC was placed under the control of an isopropyl β-D-1-thiogalactopyranoside (IPTG)-inducible promoter. This strain was unable to grow when streaked onto media lacking IPTG, while it grew robustly on plates supplemented with IPTG. Further, after 3 h of culturing in liquid media without IPTG, the bacteria ceased to grow. By 5 h, bacterial viability decreased dramatically (nearly 13-fold) compared to the LptD-expressing strain. These experiments indicated that LptD is likely essential for GC viability. To test whether NGO1344, NGO1985, NGO2111, NGO2121, NGO2139, and TamB play functions in the integrity of the GC cell envelope, the individual clean deletion mutants were constructed in strain FA1090 and spotted on plates supplemented with various compounds. Although the loss of these proteins had no effect on bacterial growth under permissive conditions, the loss of NGO1985 resulted in a severe growth defect in the presence of bile salts, polymyxin B, Tween 20, SDS, urea, and chloramphenicol. Further, these phenotypes could be completely reversed by complementation with an IPTG-inducible version of the *ngo1985* gene. Additionally, NGO2121 exhibited reduced growth in the presence of bile salts and polymyxin B. These proteins, identified by quantitative proteomic approaches, appear to provide an important function in maintaining cell membrane integrity and, as such, are promising targets for development of new therapeutic interventions against GC (Zielke et al., 2014).

To continue this research endeavor, we went on to determine the ubiquitously and specifically expressed cell envelope proteins of GC FA1090 challenged with host-relevant environmental stimuli: oxygen availability, iron deprivation, and the presence of human serum (Zielke et al., 2015). A myriad of novel proteins have been identified. Our initial characterization of five novel vaccine candidates that were ubiquitously expressed under these different growth conditions demonstrated that BamA, LptD, TamA, NGO2054, and NGO2139 were surface exposed and produced bactericidal antibodies that cross-reacted with a panel of diverse GC isolates. These promising results strongly suggest that the proteomics-driven approach will provide a foundation for the development of anti-GC vaccine(s), which would be the ideal way to prevent gonorrhea. Finally, to promote the utilization of the newly identified proteins and the knowledge of the GC subproteome dynamics among the scientific community, our entire data sets from all these investigations were made publicly available via the ProteomeXchange Consortium^4^, the PRIDE partner repository ProteomeXchange with the identifiers PXD000549 and PXD001944.

## Future Directions

Although proteomic approaches have revealed a multitude of information on the physiology of GC, to formulate an effective vaccine, more information needs to be gathered about the way its surface proteins interact with host cells during an infection. Ideally, the proteome of clinical samples that have never been subcultured should be examined to determine which proteins are expressed during different stages of infection. This approach presents a significant technical challenge to overcome, as sufficient bacterial material must be collected for a quantitative proteomic analysis. In our investigation of the cell envelope and MV proteome, we collected material from 1 liter of culture (Zielke et al., 2014). Although other proteomic studies have used less material, including 5 milliliters of culture (Wu et al., 2010) or bacteria harvested from 20 plates (Anonsen et al., 2012; Perez-Cruz et al., 2015) collecting this amount of sample from a patient is not feasible; more sensitive MS analyzers or alternate methods of sample enrichment will be required. These studies will give insight into the proteomic adaptations the bacteria undergo to establish and maintain infection. Information collected from proteomic studies of clinical samples and host tissue culture can help further drive vaccine development and have the potential to aid in the discovery of stably expressed protein targets of antimicrobial agents with novel modes of action. Integration of multiple approaches, including public access to on-line raw data is essential if there is to be a sense of participation across the biomedical research community.

An interesting analysis technique was recently pioneered by Altindis et al. (2015). Termed “protectome analysis,” their technique searches for vaccine candidate proteins in proteomic datasets by identifying proteins with structural or functional features in common with proteins known to provide protection. This analysis tool could be used in combination with other proteomic studies to immediately identify proteins expressed during infection with the potential to provide protection against reinfection.

As more information is deposited into proteomic databases, the utility of proteomic approaches to identify GC in clinical samples will increase. For example, in cases where the molecular determinant of antimicrobial resistance is known, proteomic approaches have the potential to immediately recognize the protein modification(s) that result in antimicrobial resistance, a feat that is not possible with current NAAT identification techniques.

Finally, proteomic investigations of multidrug resistance strains can reveal the mode of drug action, as well as the pathway(s) the bacterium uses to resist multiple antibiotics. One mystery that proteomic approaches may be able to solve is the identity of “Factor X,” an unknown determinant of penicillin and cephalosporin resistance that is non-transformable and therefore is difficult to study with typical genetic methods (Unemo and Shafer, 2014).

## Conclusion

Developing novel vaccines or antimicrobial agents is critical in the face of growing antibiotic resistance, and global and quantitative proteomic approaches have begun to reveal potential targets in the fight against GC. Proteomic approaches are ideal for the discovery of vaccine candidate proteins, as well as protein targets for the development of novel antimicrobial agents. Qualitative proteomic studies revealed the GC defense response to spectinomycin, while quantitative proteomics have demonstrated bacterial adaptations to conditions encountered in the host, including oxidative stress, anoxia, iron deprivation, and the presence of human serum. Proteomics have also been recently adapted to identify GC in clinical samples, which can expedite treatment. Importantly for vaccine development, stably expressed proteins have been identified through high-throughput examinations of cell envelopes and naturally released MVs. These proteomic studies will act as starting points for studies into structural vaccinology, protein–protein interactions, and GC physiology, and have already given new insights into ways to combat this important, difficult to treat pathogen.

### Conflict of Interest Statement

The authors declare that the research was conducted in the absence of any commercial or financial relationships that could be construed as a potential conflict of interest.

## Footnotes

1 http://www.broadinstitute.org/

2 http://pubmlst.org/neisseria/

3 http://www.broadinstitute.org/annotation/genome/neisseria_gonorrhoeae/MultiHome.html

4 http://www.proteomexchange.org
